# The Prevalence of Thyroid Autoimmunity in Children with Developmental Dyslexia

**DOI:** 10.1155/2021/7656843

**Published:** 2021-02-08

**Authors:** Roberta Degrandi, Flavia Prodam, Giulia Genoni, Giorgio Bellomo, Gianni Bona, Mara Giordano, Simonetta Bellone, Alice Monzani

**Affiliations:** ^1^Division of Pediatrics, Department of Health Sciences, Università del Piemonte Orientale, 28100 Novara, Italy; ^2^Department of Health Sciences, Università del Piemonte Orientale, 28100 Novara, Italy; ^3^Pediatric and Neonatal Intensive Care Unit, Maggiore della Carità University Hospital, 28100 Novara, Italy; ^4^Clinical Chemistry Laboratory, Department of Health Sciences, Università del Piemonte Orientale, 28100 Novara, Italy; ^5^Laboratory of Human Genetics, Department of Health Sciences, Università del Piemonte Orientale, 28100 Novara, Italy

## Abstract

**Methods:**

We enrolled pediatric subjects with developmental dyslexia and, as a control group, healthy age- and sex-matched subjects without developmental dyslexia. Thyroid function was evaluated in subjects with developmental dyslexia measuring serum concentrations of thyroid-stimulating hormone (TSH), free triiodothyronine (fT3), and free thyroxine (fT4). Thyroid autoimmunity was evaluated in all subjects measuring antithyroid peroxidase (TPO-Ab) and antithyroglobulin (TG-Ab) antibodies. In subjects with developmental dyslexia, thyroid ultrasonography (US) was also performed.

**Results:**

We enrolled 51 subjects with developmental dyslexia (M : F = 39 : 12, mean age 12.4 ± 9 years) and 34 controls (M : F = 24 : 10, mean age 10.8 ± 4 years). TPO-Ab positivity was significantly higher in subjects with developmental dyslexia compared to controls (60.8% vs. 2.9%, *p* < 0.001), while no significant difference was found in TG-Ab positivity (16% vs. 5.8%). Thyroid US performed in 49 subjects with developmental dyslexia revealed a thyroiditis pattern in 60%.

**Conclusions:**

We found an extremely high prevalence of thyroid autoimmunity in children with developmental dyslexia. Further studies are needed to confirm our observations, but our findings may change the approach to this disorder and eventually lead to a systematic determination of thyroid autoimmunity in children with developmental dyslexia.

## 1. Introduction

Developmental dyslexia is defined as a persistent reading difficulty not explained by sensorial or cognitive deficits, lack of motivation, or inadequate education [1, 2]. Its prevalence in school-aged children is about 5-17% [2, 3].

An association between developmental dyslexia and autoimmune disorders has been postulated in previous studies. This supposition started from the observation of a higher prevalence of both immune disorders and learning disabilities in left-handed subjects, the so-called Geschwind-Behan-Galaburda hypothesis [4, 5]. It was hypothesized that, if left-handedness is more frequent in patients with autoimmune diseases as well as developmental disorders are more frequent in left-handed people [6], then an association might exist also between autoimmune diseases and developmental disorders. Only a few previous studies reported a high prevalence of autoimmune diseases in subjects with developmental dyslexia [7, 8], but the actual prevalence of thyroid autoimmunity was not assessed yet.

The aim of our study was to evaluate the prevalence of thyroid autoimmunity in pediatric patients with developmental dyslexia.

## 2. Materials and Methods

### 2.1. Subjects

In the period June 2015–January 2017, we enrolled pediatric subjects with developmental dyslexia referred by patients' associations. Inclusion criteria were the presence of a certified diagnosis of developmental dyslexia, a normal intelligence according to Wechsler Intelligence Scale for Children (general intelligence quotient ≥ 70) [9, 10], and an age range of 6-20 years. The diagnosis of dyslexia was based on an extensive examination including neurological, psychological, and phonological capabilities, performed less than a year before being included in the present study. Reading abilities were evaluated using the Text Comprehension and Decoding Test [11], the word subtest from the Battery for the Evaluation of Developmental Dyslexia and Dysgraphia [12], and the Word and Nonword Test [13] for Italian-speaking children. Dyslexia was defined if reading abilities scores were 2 SD below the normal scores. Exclusion criteria were other coexisting chronic or genetic diseases or the use of drugs altering thyroid function. Data about dominant hand were collected to define right- or left-handedness.

As a control group, healthy age- and sex-matched subjects without developmental dyslexia were enrolled. Control subjects were outpatients of the Surgery Unit of our hospital, who underwent a blood sample collection before a surgical intervention for the treatment of minor diseases (i.e., phimosis, hydrocele, inguinal hernia, etc.). To be enrolled, they should have no history of chronic disease and receive no treatments.

The study protocol was approved by the local Ethics Committee (CE 161/15), and written informed consent was collected from the parents or legal guardians of all subjects and patients themselves, where appropriate.

### 2.2. Thyroid Evaluation

All the enrolled subjects underwent a blood sample collection in the morning after an overnight fast. Thyroid function was evaluated in subjects with developmental dyslexia measuring serum concentrations of thyroid-stimulating hormone (TSH), free triiodothyronine (fT3), and free thyroxine (fT4). Thyroid autoimmunity was evaluated in all subjects measuring antithyroid peroxidase (TPO-Ab) and antithyroglobulin (TG-Ab) antibodies. Serum TSH, fT3, fT4, TPO-Ab, and TG-Ab levels were assessed in the Clinical Chemistry Laboratory of our University Hospital by direct chemiluminescent technology with acridinium ester as a label and paramagnetic particles as a solid phase (ADVIA Centaur CP Immunoassay System; Siemens Healthcare Diagnostics, Deerfield, IL). Sensitivity was 0.010 *μ*IU/mL for TSH, 0.2 pg/mL for fT3, 0.1 ng/dL for fT4, 28 IU/mL for TPO-Ab, and 15 IU/mL for TG-Ab assay. Reference values were 0.450–3.500 *μ*IU/mL for TSH, 2.30–4.20 pg/mL for fT3, and 0.89–1.76 ng/dL for fT4. TPO-Ab and TG-Ab titers higher than 60 IU/mL were considered positive, according to our laboratory range.

In subjects with developmental dyslexia, thyroid ultrasonography (US) was also performed by a single trained operator, who was not aware of the results of laboratory tests. Thyroid size and morphology were evaluated using a high-resolution 7-13 MHz linear transducer, with the subjects sitting and their necks slightly extended. The volume (milliliters) of each lobe of the thyroid gland was calculated using the following formula: 0.479 × depth × length × width. Thyroid volume was defined as enlarged according to the reference values for sex and body surface area [14]. Thyroid tissue echogenicity was evaluated in a longitudinal scan of the thyroid lobes by a standardized comparison with the echogenicity of the adjacent muscles and categorized as normal, decreased, or increased compared to them. The presence of multiple hypoechoic foci or patches scattered throughout an otherwise normal echogenic gland or a gland with diffuse hypoechogenicity, compared to the anterior strap muscle, has been considered as an ultrasonographic pattern of thyroiditis.

### 2.3. Statistical Analysis

Data were expressed as mean ± SD and range, for continuous variables, and as number and percentage (%), for categorical variables. Comparisons between the prevalence of positive antithyroid antibodies in subjects with developmental dyslexia and control subjects were performed using a chi-squared test or Fisher test, as appropriate. A *p* value of <0.05 was considered statistically significant. All analyses were performed using SPSS version 21.0 (IBM, New York, NY, USA).

## 3. Results and Discussion

We enrolled 51 subjects with developmental dyslexia (M : F = 39 : 12), aged 7.0-19.2 years (mean 12.4 ± 9 years) and 34 healthy control subjects (M : F = 24 : 10), aged 6.0-18.0 years (mean 10.8 ± 4 years). Two out of 51 subjects with developmental dyslexia refused their consent to the US examination.

We collected data on the dominant hand in 46 of 51 subjects: 40 were right-handed (87%) and 6 left-handed (13%).

### 3.1. Thyroid Function

TSH, fT3, and fT4 levels were measured in subjects with developmental dyslexia. Mean TSH was 2.266 ± 1.003 *μ*IU/mL (range 0.718–5.478 *μ*IU/mL). Mean fT3 and fT4 were, respectively, 3.86 ± 0.56 pg/mL (range 1.30–5.20 pg/mL) and 1.25 ± 1.6 ng/dL (range 0.91–1.60 ng/dL). Subclinical hypothyroidism was found in 5 subjects (9.8%). None had overt hypothyroidism. TSH and thyroid hormones levels were similar in males and females.

### 3.2. Antibody Profiling

**Figure 1:**
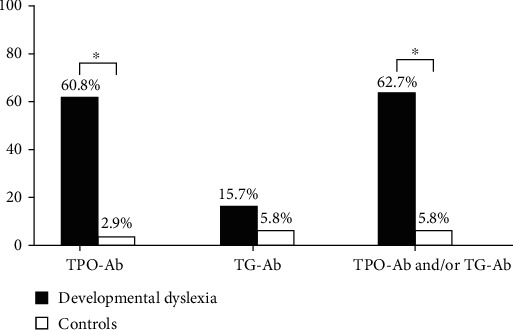
Prevalence of subjects with positive antithyroid peroxidase (TPO-Ab) and/or antithyroglobulin (TG-Ab) antibodies in cases (subjects with developmental dyslexia) and controls.

Antithyroid antibodies were measured both in subjects with developmental dyslexia and in controls. In subjects with developmental dyslexia, TPO-Ab values ranged between 28 and 1300 IU/mL, and TG-Ab values between 30 and 2500 IU/mL. TPO-Ab positivity was found in 31 (60.8%) and TG-Ab positivity in 8 subjects with developmental dyslexia (16%). Overall, 32 subjects with developmental dyslexia (63%) had at least one positive antibody, and 7 subjects (14%) had both positive antibodies. The prevalence of positive antithyroid antibodies was compared to that found in age- and sex-matched healthy controls. TPO-Ab positivity was found in 1 control (2.9%) and TG-Ab positivity in 2 control subjects (5.8%). Overall, 2 control subjects (5.8%) had at least one positive antibody and 1 control subject (2.9%) had both positive antibodies. TPO-Ab positivity was significantly higher in subjects with developmental dyslexia than in controls (*p* < 0.001). The prevalence of subjects with developmental dyslexia having at least one antibody positivity was significantly higher than that of controls (*p* < 0.001) (Figure 1). The prevalence of positive antithyroid antibodies was not different according to gender or right/left handedness.

### 3.3. Thyroid Ultrasound

Thyroid US was performed in 49 subjects with developmental dyslexia: 29 of them (60%) presented a thyroiditis pattern. Thyroid nodules were found in 2 subjects (4%).

Fifteen subjects (29%) with positive antithyroid antibodies had a negative ultrasound, whereas 14 subjects (27%) with a suggestive ultrasound did not show antibody positivity.

The presence of thyroid autoimmunity (defined by antibody positivity and/or thyroiditis pattern at ultrasound) was observed in 46 subjects (90%). The prevalence of thyroid autoimmunity was not different according to gender or right/left handedness.

## 4. Discussion

In the present study, we found a striking elevated prevalence (over 90%) of thyroid autoimmunity, defined as the presence of positive antithyroid antibodies and/or US pattern suggestive of thyroiditis, in children with developmental dyslexia. Almost two-thirds of them had positive antithyroid antibodies, represented in 60.8% of cases by elevated TPO-Ab. This rate was significantly higher than that found in age- and sex-matched healthy subjects. The previously reported prevalence of TPO-Ab or TG-Ab positivity in healthy children ranges from 2.5% to 7% [15–19]. Taubner et al. [20] studied a wide cohort of 841 neonates, children, and adolescents (aged 0-20 years) with no evidence of thyroid disease. They found that TPO-Ab and TG-Ab were relatively raised in the first year of life and that a secondary rise in both TPO-Ab and TG-Ab can be seen in girls but not in boys in puberty and adolescence. Notably, we found an extremely high prevalence of thyroid antibodies positivity in subjects with developmental dyslexia, across all the studied ages and without differences according to gender distribution.

The prevalence of thyroid autoimmunity became even higher including subjects with a thyroid US pattern suggestive of thyroiditis. In our series, 14 subjects presented a suggestive thyroid ultrasound pattern without circulating antithyroid antibodies. As stated in a precedent study [21], it is possible that ultrasound positivity precedes antibody positivity, allowing early identification of thyroid autoimmunity. Indeed, subjects with thyroid hypoechogenicity or inhomogeneity may be considered at greater risk of developing autoimmune thyroiditis, regardless of the presence of antithyroid antibodies. In our sample, we also found 15 subjects with circulating antibodies with a negative thyroid ultrasound. We could assume that in these children, circulating antibodies have not caused thyroid damage yet.

Elevated prevalence of immune disorders in subjects with developmental dyslexia was previously reported, even if thyroid autoimmunity was not specifically addressed. In the 80s-90s, Hugdahl et al. [8] investigated the presence of immune disorders (allergies, eczema, asthma, and uveitis), autoimmune disorders (Crohn disease, ulcerative rectocolitis, celiac disease, type 1 diabetes, thyroid autoimmune disease, rheumatoid disorders, and neuromuscular disorders), and left-handedness in 105 dyslexic children and 105 age- and sex-matched controls. A higher frequency of immune and autoimmune disorders in dyslexic subjects (53%) compared to nondyslexic subjects (25%) was observed. However, the presence of immune or autoimmune disorders was assessed only on the basis of self-administered questionnaires, and the prevalence of each condition was not reported [8]. Similarly, Pennington et al. [7] studied autoimmune diseases (early-onset rheumatoid arthritis, multiple sclerosis, ulcerative colitis, systemic lupus erythematosus, Hashimoto's thyroiditis, Graves' disease, type 1 diabetes, myasthenia gravis, uveitis, and dermatomyositis), allergies (asthma, food allergies), and neuropsychiatric disorder prevalence in 87 dyslexic subjects and 86 nondyslexic subjects. They found a significantly higher prevalence of autoimmune diseases in the dyslexic group (10% vs. 1.5%). However, even in this study, an investigation of the autoimmune profile was not carried out, and it simply relied on patient questionnaires [7]. Moreover, from a different perspective, Wood and Cooper administered reading tests in 74 men with autoimmune thyroid diseases compared to 24 controls affected by nonautoimmune thyroid diseases (thyroid cancer, goiter) [22]. In the former, they found stuttering, inversions, unreadable handwriting, spelling mistakes, or dyslexia with a greater frequency. Nonetheless, a proper diagnosis of dyslexia was not documented.

Conversely, Gilger et al. found no association between developmental reading disability and immune disorders (early-onset rheumatoid arthritis, multiple sclerosis, thyroid disorders, ulcerative colitis, systemic lupus erythematosus, diabetes mellitus, myasthenia gravis, uveitis, and dermatomyositis). But in particular, the presence of thyroid autoimmunity was assessed only by a survey and not by antibody testing [23].

To explain our results, the Geschwind-Behan-Galaburda hypothesis regarding the presumed association between left-handedness, developmental dyslexia, and immunity is undoubtedly fascinating [4]. However, the theory used to explain this association is open to criticism. In fact, they assumed that testosterone acts on both the cerebral tissue and thymus gland, causing the development of dyslexia and left-handedness on the one hand and autoimmunity on the other. If we assume this theory as true, autoimmune diseases should be more frequent in males, but it is known that autoimmunity is more frequent in females [24]. Sex distribution in our sample reflected the characteristic pattern of developmental dyslexia, which is more common in males (with a male : female ratio of 3.5-4 : 1). However, the role of androgens in utero cannot be ruled out by our study. Furthermore, despite the high prevalence of thyroid autoimmunity in our population, the prevalence of left-handedness was similar to that reported in the general population [25] and not increased, in contrast with Geschwind's hypothesis. Translational studies are needed to explain our findings and whether the relationship between thyroid autoimmunity and dyslexia is also causative.

Most of the enrolled subjects were euthyroid, with less than 10% of dyslexic subjects showing subclinical hypothyroidism. Nonetheless, the prevalence of subclinical hypothyroidism in our cohort was higher than that reported in pediatric age, being about 2%. This finding is consistent with the high prevalence of thyroid autoimmunity found in our cohort, and it is known that the presence of elevated antithyroid antibodies may be suggestive of the persistence of subclinical hypothyroidism over time or even of the progression to overt hypothyroidism [26].

A limit of our study was that family history for thyroid disorders was not investigated. However, the possible influence of positive family history for thyroid diseases could hardly explain such a higher prevalence of thyroid autoimmunity in our children with developmental dyslexia, as it is known that only about 30% of children with Hashimoto's thyroiditis have at least one first-degree relative with thyroid diseases [27]. At the same time, it is improbable to assume that thyroid disorders would cluster in families of subjects with developmental dyslexia more frequently than in controls, without recognizing a certain link between dyslexia and thyroid dysfunctions.

A second limit is that US evaluation was performed only in subjects with developmental dyslexia and not in controls, so that we cannot exclude that some healthy controls would have displayed thyroid US alterations. However, the prevalence of thyroid autoimmunity merely assessed on the basis of positive antibodies is much higher in dyslexic subjects than in controls that it is improbable that the findings at US evaluation in controls would decrease the strength of our results.

Furthermore, pubertal status was not evaluated in our patients, preventing the detection of differences in thyroid autoimmunity prevalence according to puberty.

Moreover, only thyroid autoimmunity was evaluated, but the current results pave the way to the investigation of other autoimmune profiles in our cohort of dyslexic subjects, in particular antinuclear antigen, rheumatoid factor, and celiac antibodies.

Finally, it would be informative to know the actual value of TPO-Ab, but it is a limit that could not be overcome, as the overflow values are presented just as >60 IU/mL according to the kit used in our lab.

## 5. Conclusions

In conclusion, the detected association between thyroid autoimmunity and dyslexia may change the approach to children affected by developmental dyslexia. From a clinical point of view, asymptomatic children with circulating antibodies and characteristic ultrasound patterns are at greater risk of developing thyroid dysfunction. Although further studies are required to confirm our findings, they may eventually lead to a systematic determination of thyroid antibody profiling and thyroid function tests in dyslexic children. From a pathophysiologic point of view, this association may suggest a relationship between the pathogenesis of thyroid autoimmune disorders and developmental disorders, even if our findings do not claim to demonstrate a causative link between thyroid autoimmunity and developmental dyslexia, but just to describe an unexpected high association between them. In this regard, further studies are needed to explain this association and to evaluate in subjects with developmental dyslexia the prevalence of other widespread autoimmune diseases, such as type 1 diabetes mellitus and celiac disease, which are often in comorbidity with thyroid autoimmune disorders. It still remains a challenge to understand if autoimmunity is comorbidity or if it is implicated in the pathophysiology of developmental dyslexia.

## Data Availability

The data used to support the findings of this study are available from the corresponding author upon request.
